# Magnitude and associated factors of postpartum morbidity in public health institutions of Debre Markos town, North West Ethiopia

**DOI:** 10.1186/s40748-018-0086-0

**Published:** 2018-10-03

**Authors:** Asmare Talie, Abere Yekoye, Megbaru Alemu, Belsity Temesgen, Yibeltal Aschale

**Affiliations:** 1 Department of Midwifery, College of Health Sciences, Debre Markos University, Debre Markos, Ethiopia; 2 Department of Midwifery, College of Health Sciences, MekelleUniversity, Mekelle, Ethiopia; 3 Department of Immunology, Microbiology and Parasitology, College of Health Science, Bahir Dar University, Bahir Dar, Ethiopia; 4 Department of Medical Parasitology, College of Health Sciences, Debre Markos University, Debre Markos, Ethiopia

**Keywords:** Postpartum morbidity, Health institution, Magnitude, Debre Markos, Ethiopia

## Abstract

**Background:**

Postpartum maternal morbidity is maternal illness that occurs after one hour of expulsion of placenta up to six weeks of childbirth. Though the true burden of this problem is not well known estimates of WHO, UNICEF and UNFPA showed that 1.4 million women experience acute obstetric morbidity annually. Knowledge of magnitude and predicting factors postpartum morbidity is central to understand the extent of the problem and will help as a cornerstone in designing and implementing better preventive strategies.

**Objectives:**

To assess the magnitude and factors associated with postpartum morbidity in public health institutions in Debre Markos town.

**Method:**

Institutional based cross sectional study was conducted in Debre Markos town public health institutions by reviewing delivery charts, delivery records and reporting log books. Total deliveries in each health institution in the previous year were identified and number of records to be included from each institution was determined by probability proportion to size. Systematic sampling technique was employed to select 308 charts for review. Data was collected by trained midwifes using structured checklist; entered by epi info and analyzed using SPSS 20. To present findings descriptive statistics using frequencies, charts and figures were used accordingly. Finally binary and multiple logistic regressions were performed to identify predicting factors.

**Results:**

The magnitude of postpartum morbidity was found to be 101(32.8%). Divorced/widowed women [AOR = 10.920, 95% CI: (2.168, 54.998)], women who didn’t have ANC follow up [AOR = 3.710, 95% CI: (1.749, 7.870)], abnormal labour [AOR =3.496, 95% CI: (1.69, 7.22)], women delivered by doctor [AOR =0.111, 95% CI: (0.027, 0.454)] and women who were not attended postpartum visit [AOR =0.088, 95% CI: (0.040, 0.194)] were the factors associated with postpartum maternal morbidity.

**Conclusion:**

Maternal morbidity in Debre Markos health institution was found to be major maternal health issue. Being divorced/widowed, absence of ANC visit, intrapartum abnormalities, delivery attended by skilled professionals and no post-partum visit were important predictors of maternal postpartum morbidity.

## Background

Postpartum maternal morbidity is maternal illness that occurs after one hour of expulsion of placenta up to six weeks of childbirth. It is complex, with multiple causes, duration ranging from acute to chronic, severity from transient to permanent with different diagnosis and treatment options [1]. It is major maternal health problem affecting both developed and developing countries on human, social and economic development and associated with poor or nonexistent medical care during labor and after birth [2].

Globally maternal mortality ratio (MMR) declined from 400 maternal deaths per 100,000 live births in 1990 to 210 in 2010, but in Ethiopia MMR increased from673/100,000 live births to 676/100,000 live births [2]. Greater than 60% of maternal deaths worldwide occurred in the postpartum period; of this 45% of postpartum deaths occurred within 1 day of delivery. In developing countries 80% of postpartum deaths caused by obstetric factors which occurred within 1 week of delivery [3].

In developing countries 15-20million women develop disability each year as a result of child birth complications. This is due to lack of skilled birth attendants; most women who experience complications do not receive adequate medical attention to avert serious illness due to failures to recognize the warning signs of complications or fear of poor treatment or high fees at health facilities [4].

Literatures on maternal morbidity showed that severe anemia, delivery at home, low socioeconomic status, and Para three or more are contributing factors for post-partum morbidity in India [5]. In sub-Saharan Africa postpartum morbidity is due to obstructed labour (4%), hypertension disorder (9%), unsafe abortion (4%), infection (10%) and hemorrhage (34%) are causes of postpartum morbidity sub-Saharan Africa [6].

Despite the fact that postpartum morbidity is a serious maternal health problem with lots of contributing factors; very little is known about its magnitude and influencing factors in Ethiopia. Therefore this study aims to close this gap by determining the magnitude and ascertain contributing factors that will help as a foundation in designing preventive strategies.

## Methods and materials

### Study area and period

The study was conducted in Debre Markos town; a capital city of East Gojam zone located about 300 from Addis Ababa, and 276 km from the Amhara region capital, Bahir Dar. According to the town finance and economic office report, the total population of the town is 101,582 (Male = 49,775, Female 52,806). Regarding public health institution there are 7 health posts, 3 health centers one family guidance association clinic (FGA) and one referral hospital in the town. The gynecology and obstetrics unit of this referral hospital have 20 midwives, two gynecologists and two emergency surgeons with greater than 100 deliveries per month. In the three health centers there are 28 nurses, 11 health officers and 8 midwives with average of 20 deliveries per month in each health center.

### Study design

Institution based retrospective cross sectional study was conducted in public health institutions.

### Target, source and study population

The target population involves all delivery records in public health institution in Debre Markos town and records of postpartum women who received care in public health institutions of Debre Markos town in the previous year were the source population. The study population contains maternal charts which fulfill inclusion criteria and selected systematically from the source population.

### Sample size determination and sampling procedure

Sample size was computed using single population proportion formula by using the following assumption the prevalence (P) of postpartum morbidity as 23.6% [15] with 95% CI and 5% marginal error (d) which gives sample size of 280. By adding 10% contingency for incomplete maternal records the final sample size became 308**.**Total number of deliveries in each public health institution over the previous one year was identified and population proportion to size was done to determine number of records to be included from each institution. Finally systematic sampling technique using sampling interval of six was used to select records from each institution for review.

### Data collection tool and procedure

Eight data collectors who had previous experience in similar assignments participated in data collection by reviewing of mother’s charts; delivery records of labour ward and reporting log book. Structured checklist prepared in English was used to collect data after pretested in 5% of maternal cards which were not included in final sample size. The checklist included Socio-demographic factors, maternal health service and obstetric factors of postpartum morbidity. Two data collection supervisors supervised the data collection process regularly.

### Variables

#### Dependent variable

Magnitude of postpartum morbidity.

#### Independent variable

Socio demographic variables: age, marital status, education, place of residence, ethnicity; Variables related to Maternal Health Service: ANC, place of delivery, birth attendants, number of ANC visit, postpartum care and obstetric factors: obstetric complications, desire for recent pregnancy, maternal HIV status, mode of delivery, Iron supplementation in last pregnancy, duration of labour and parity.

### Data entry and analysis

Data was coded and entered to computer using epi info 7 and exported to SPSS version 22 for analysis. Descriptive statistics was used summarize data. All potential variables were entered bivariate logistic regression and those variables with *p* value < 0.2were included in multiple logistic regression model to identify predicting factors. Strength of statistical association was measured by AOR with 95% confidence interval and *p*- value< 0.05 was used to determine statistical significance.

## Results

### Socio- demographic characteristics

In this study, a total of 308 postpartum women cards were reviewed with 100% response rate. Ninety four (30.5%) were aged between 25 and 29 years. Majority (86.4%) of mothers were married. On the other hand most (95.8%) were Amhara by ethnicity followed by 8 (2.6%) Tigre and 51.6%). (Table 1).

**Table 1:** Distribution of women by their Socio-demographic characteristics at Debre Markos town public health institutions, North West Ethiopia, April 1, 2015 to March 30, 2016 (*n*=308)

Variable	Frequency (*n*)	Percentage (%)
Age of the mother (in years)		
15-19	30	9.8
20-24	86	27.9
25-29	94	30.5
30-34	48	15.6
>=35	50	16.2
Place of residence		
DebreMarkos town	140	45.5
Out of DebreMarkos town	168	54.5
Maternal Education		
No education	141	45.8
Literate	167	54.2
Marital Status		
Unmarried	26	8.4
Married	266	86.4
Divorced/widowed	16	5.2

### Obstetric (pregnancy and intrapartum) characteristics

During their most recent pregnancy, 230 (74.7%) of women had ANC follow up of whom only 79 (34.3%) had four ANC visits. Majority of them 210 (68.2%) received iron and folic acid supplementation during pregnancy. Regarding maternal HIV status, 245 (79.5%) were negative followed by 60 (19.5%) and 3 (1%) with unknown and seropositive status respectively. Majority, 193 (62.7%) women were delivered by midwife. The most common complication women encountered during their most recent pregnancy was infection 31 (26.5%) followed bay mal presentation 29 (24.8%) (Table 2).

**Table 2:** Distribution of women by their obstetric history in DebreMarkos town public health institutions, North West Ethiopia, April 1, 2015 to March 30, 2016 (*n*=308)

Variable	Frequency (*n*)	Percentage (%)
Number of ANC visit		
1	64	27.8
2-3	77	33.5
4	79	34.3
>4	10	4.4
Type of complication during this pregnancy		
Anemia	16	13.7
APH	17	14.5
preeclampsia/eclampsia	13	11.1
Infection	31	26.5
Mal-presentation	29	24.8
Other	11	9.4
Place of delivery		
Health center	130	42.2
Hospital	144	46.8
Home	34	11.0
Birth attendant		
Doctor	47	15.3
Midwife	193	62.7
Nurse/health officer	34	11.0
Unskilled	34	11.0
Mode of delivery		
SVD	206	66.9
instrumental delivery	41	13.3
SVD +episiotomy	31	10.1
Cesarean section	30	9.7

### Postpartum morbidity

**Fig. 1:**
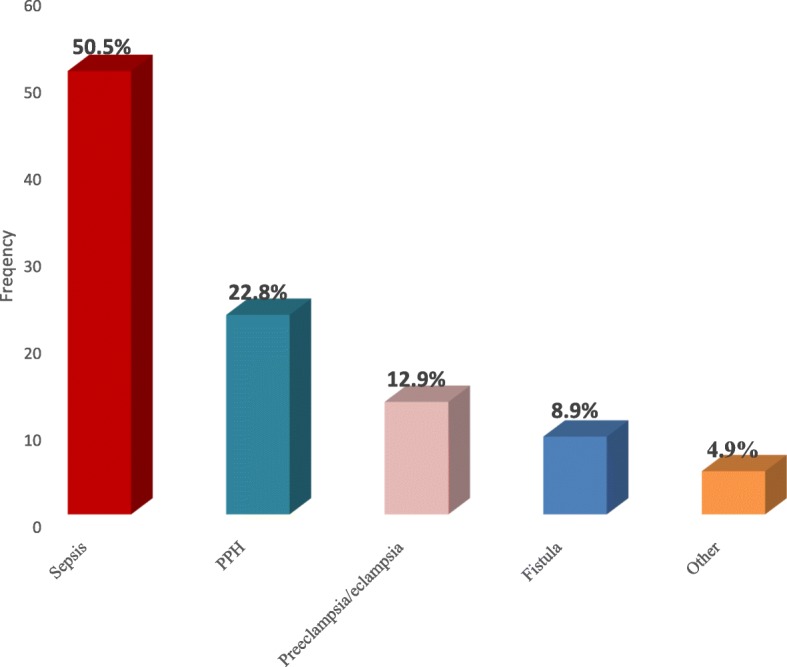
Types of Postpartum Maternal Morbidities at Debre Markos Town Public Health Institutions North West Ethiopia, April 1, 2015 to March 30, 2016

The magnitude of postpartum morbidity was found to be 101 (32.8%). Over all morbidities sepsis had the highest prevalence 51 (50.5%) (Fig. 1). The main causes of postpartum sepsis were genitor-urinary tract infection accounted 29(56.9%), wound infections 16 (31.3%) like C/S 6 (37.4%), episiotomy 5 (31.3%), perineal tear 5 (31.3%) and breast complications 6 (11.8%).

### Factors associated with postpartum morbidity

Variables considered for multivariate logistic regression analysis were those with a *p*-value< 0.2 in bivariate analysis and these were place of residence, marital status, ANC service, maternal education, parity, iron supplementation in pregnancy, maternal HIV status, abnormal labour, mode of delivery, birth attendant, past postpartum morbidity and postpartum visit.

After controlling for confounding variables using multiple logistic regression; marital status, ANC service, birth attendant, abnormal labour and postpartum visit showed significant and independent association with postpartum maternal morbidity.

Divorced/widowed women had eleven times higher odds of developing postpartum complications than married women [AOR = 10.920, 95% CI: (2.168, 54.998)]. Women who didn’t obtain ANC visit were 3.71 times more likely to develop postpartum morbidity when compared to their counterparts [AOR = 3.710, 95% CI: (1.749, 7.870)]. Women who had labor abnormality in their last delivery were 3.5 times higher odds to develop postpartum morbidity than those who didn’t have abnormal labour [AOR =3.496, 95% CI: (1.69, 7.22)].

Women delivered by doctor around 89% less likely develop postpartum complications than women delivered by unskilled birth attendants [AOR =0.111, 95% CI: (0.027, 0.454)]. Similarly, women who delivered by nurse/health officer were 94% less likely developing postpartum morbidity than women delivered by unskilled birth attendants [AOR =0.058, 95% CI: (0.009, 0.361)]. More over; those mothers who hadn’t attend postpartum visit were 91.2% less likely to have postpartum morbidity compared to mothers who had postpartum visit [AOR =0.088, 95% CI: (0.040, 0.194)] (Table 3).

**Table 3:** Bivariate and multiple logistic regression analysis of factors associated with postpartum morbidity in DebreMarkos Town Public Health Institutions, April 1, 2015 to March 30, 2016 (*n*=308)

Variables	Bivariate Analysis			Multivariate Analysis			
	COR	95%CI		*P*-value	AOR	95%CI	
		Lower	upper			Lower	Upper
Residence							
DebreMarkos	0.304	0.181	0.510	0.131	0.572	0.277	1.181
Out of DebreMarkos	1				1		
Marital Status							
Married	1				1		
unmarried	0.673	0.261	1.738	0.538	0.684	0.204	2.292
Divorced/widowed	9.724*	2.698	35.045	0.004	10.920	2.168	54.998**
Maternal education							
No education	1.897	1.172	3.070	0.867	0.940	0.455	1.943
Literate	1				1		
Parity							
0	0.243	0.076	0.781	0.061	0.157	0.023	1.089
1	0.313	0.154	0.637	0.282	0.552	0.187	1.629
2-4	0.413	0.198	0.860	0.826	1.136	0.362	3.567
>=5	1				1		
ANC follow up							
No	6.268*	3.589	10.945	0.001	3.710	1.749	7.870**
Yes	1				1		
Iron supplementation during pregnancy							
No	4.256	2.547	7.113	0.213	0.443	0.123	1.597
Yes	1				1		
Maternal HIV status							
Negative	1				1		
Positive	1.275	0.114	14.293	0.863	1.349	0.044	41.095
Un known	2.727	1.530	4.859	0.705	1.185	0.493	2.851
Birth Attendant							
Midwife	0.049	0.017	0.146	0.050	0.284	0.081	1.000
Doctor	0.069*	0.021	0.230	0.002	0.111	0.027	0.454**
Nurse/health officer	0.013*	0.003	0.063	0.002	0.058	0.009	0.361**
Unskilled	1				1		
Mode of delivery							
SVD	1				1		
Instrumental delivery	0.442	0.194	1.006	0.042	0.262	0.072	0.952
SVD+ episiotomy	0.437	0.172	1.114	0.173	0.382	0.096	1.523
C/S	1.594	0.737	3.450	0.320	0.355	0.046	2.738
Abnormal Labour							
No	1				1		
Yes	3.524*	2.142	5.796	0.001	3.496	1.692	7.223**
Past postpartum morbidity							
No	1				1		
Yes	5.695	3.167	10.242	0.340	1.582	0.617	4.057
Postpartum visit after this delivery							
No	0.082*	0.042	0.159	0.000	0.088	0.040	0.194**
Yes	1				1		

## Discussion

This study was carried out to determine magnitude of post-partum morbidity and identify predicting factors in government health institutions in Debre Markos town. Childbirth is a joyful experience for many but unfortunately it can be a difficult period bringing new problems that occurred especially in the first 24 h of child birth and many more may continue to happen lifelong in the days following child birth.

This study showed that magnitude of post-partum morbidity is 32.8%. This finding is consistent with studies done in Pakistan (34.4%), and Bangladesh (30%) [7, 8]. Similar studies in US and India reported higher magnitude of post-partum morbidity; 52 and 52.6% respectively [9, 10]. On the other hand finding of this study is higher when compared to a study done in Gondar (23.6%) [11]. This gap could be because of differences in the study population, study design and study setting. The above studies include only rural residents (study in India), in referral hospital (US) and urban residents and only ANC attendants in (Gondar).Whereas this study was done both rural and urban residents.

Sepsis (50.5%), PPH (22.8%) and hypertension (12.9%) were the three major causes of postpartum morbidity in this study. Many literatures reported conflicting results regarding causes of postpartum morbidities. Findings of this study were lower when compared to a study conducted in London (PPH 36.4%, HTN 39.4%) and higher than study in Gondar which showed sepsis (6.9%) PPH (14.8%), HTN (9%) [11, 12]. Similar to this finding is an institutional based cross sectional study conducted in India which reported sepsis contribute more than half (51%) of all causes of post-partum morbidity [13].

In this study, marital status showed a significant association with postpartum morbidity. Being divorced/widowed increase the odds of having postpartum morbidity by a factor 10.92 [AOR = 10.920, 95% CI: (2.168–54.998)]. This result is similar with the study conducted in Nigeria where married women were 72% less likely to have post-partum morbidity [14].

This study confirmed that there is a statistically significant association between ANC service during last pregnancy and occurrence of maternal postpartum morbidity. Mothers who didn’t obtain any prenatal care during pregnancy were 3.71 times more likely to develop post-partum morbidity than mothers who had ANC [AOR = 3.710, 95% CI: (1.749, 7.870)].

Consistent to this study is a study done in Pakistan which reported not having prenatal care as risk for postpartum morbidity [7].

The possible explanation might be women who attend antenatal care are screened and get early treatment of pregnancy related complications that predispose to postpartum infections; increase chance of women to deliver in health institutions and also more likely to get information from health professionals towards the prevention mechanisms of postpartum morbidity.

Women having intra partum abnormality are susceptible to postpartum infections due to long stay in health institutions, may have frequent vaginal examination; might undergo cesarean section because of (obstructed labour, failed induction/augmentation) and they will have C/S complications. This study showed that, postpartum morbidity was significantly associated & influenced by intra-partum abnormalities. Women who had abnormal labour were more likely to have postpartum morbidity than their counterparts [AOR =3.496, 95% CI: (1.69–7.22)]. Consistent findings were reported from studies done in India, Gondar, Bangladesh and Pakistan [10, 11, 15, 16].

Birth attendant had significant association with postpartum maternal complication. Women delivered by doctor had fewer odds to develop postpartum complications than women delivered by unskilled birth attendants [AOR =0.111, 95% CI: (0.027, 0.454)]. Similarly, women delivered by nurse/health officer less likely to develop postpartum morbidity than women delivered by unskilled birth attendants [AOR =0.058, 95% CI: (0.009, 0.361)]. Consistent results were found from studies done in India, Pakistan, Nigeria and Morocco [5, 7, 14, 17]. This might be related to the fact that skilled birth attendants practice aseptic technique like hand washing and antiseptic materials to provide clean delivery surface, active management of third stage of labour properly applied, utilization of antibiotics during labour and after delivery. In addition to this, mothers delivered by skilled birth attendants are more likely to get information about postpartum danger signs which will increase standard of medical care by preventing postpartum morbidity. Maternal postpartum visit is another predictor of maternal postpartum morbidity. The odds of women who didn’t have postpartum visit less likely to have postpartum morbidity when compared to mothers who had postpartum visit [AOR =0.088, 95% CI: (0.040–0.194)]. The possible reason could be majority of women came to those health institutions were when they got postpartum complications.

### Strength and limitation

#### Strength

- Included all governmental health institution in the study area.

#### Limitation of the study

- Because of lack of standard format about what variables should be documented in every maternal card, some relevant variables were not registered in the clients’ document.
- Since the study was an institution based cross sectional study, the results of the study may not show the true picture of the problem in the community.

## Conclusion and recommendations

This study confirmed that post-partum morbidity in Debre Markos town public health institutions was high. Divorced/widowed women, women who didn’t have ANC service, abnormal labour, delivery attended by Doctor/nurse/health officer and women who didn’t have postpartum visit were factors associated with maternal postpartum morbidity. This implies strategies to reduce maternal mortality and morbidity should give emphasis on access of prenatal care service, early detection and management of intra-partum abnormalities, and educating mothers on benefits of institutional delivery.
